# Phase Inversion in PVDF Films with Enhanced Piezoresponse Through Spin-Coating and Quenching

**DOI:** 10.3390/polym11071096

**Published:** 2019-06-28

**Authors:** Marco Fortunato, Domenico Cavallini, Giovanni De Bellis, Fabrizio Marra, Alessio Tamburrano, Francesca Sarto, Maria Sabrina Sarto

**Affiliations:** 1 Department of Astronautical, Electrical and Energy Engineering, Sapienza University of Rome, Via Eudossiana 18, Rome 00184, Italy; 2 Research Center for Nanotechnology Applied to Engineering of Sapienza (CNIS), SNNLab, Sapienza University of Rome, Piazzale Aldo Moro, 5, Rome 00185, Italy; 3 ENEA, Frascati Research Center, Via Enrico Fermi, 45, Frascati 00044, Italy

**Keywords:** polyvinylidene fluoride (PVDF), quenching, piezoelectric effect, piezoresponse force microscopy (PFM)

## Abstract

In the present work, poly(vinylidene fluoride) (PVDF) films were produced by spin-coating, and applying different conditions of quenching, in order to investigate the dominant mechanism of the β-phase formation. The influence of the polymer/solvent mass ratio of the solution, the rotational speed of the spin-coater and the crystallization temperature of the film on both the β-phase content and the piezoelectric coefficient (*d*_33_) were investigated. This study demonstrates that the highest values of *d*_33_ are obtained when thinner films, produced with a lower concentration of polymer in the solvent (i.e., 20 wt.%), go through quenching in water, at room temperature. Whereas, in the case of higher polymer concentration (i.e., 30 wt.%), the best value of *d*_33_ (~30 pm/V) was obtained through quenching in liquid nitrogen, at the temperature of 77 K. We believe that in the former case, phase inversion is mainly originated by electrostatic interaction of PVDF with the polar molecules of water, due to the low viscosity of the polymer solution. On the contrary, in the latter case, due to higher viscosity of the solution, mechanical stretching induced on the polymer during spin-coating deposition is the main factor inducing self-alignment of the β-phase. These findings open up a new way to realize highly efficient devices for energy harvesting and wearable sensors.

## 1. Introduction

In the last ten years, piezoelectric polymer thin films have attracted a lot of interest in the production of flexible nanogenerators, sensors, and actuators [1,2,3,4]. Poly(vinylidene fluoride) (PVDF) is one of the most interesting piezoelectric polymers for a wide range of advanced applications, from sensing to energy harvesting [1,5]. The great interest in PVDF is due to its excellent thermal stability, mechanical flexibility, low-density and unique piezoelectric and ferroelectric characteristics. PVDF exists in three main polymorphs phases (α, β, and γ). The most common crystalline phases in PVDF are the α- and the β-phase. The α-phase is an electrically inactive phase, whereas the β-phase is an electroactive and polar phase, showing the strongest ferroelectric and piezoelectric behaviour [6]. In order to increase the electroactive response of PVDF, the CH_2_–CF_2_ dipoles must be aligned and oriented along a preferential direction. Dipoles orientation is usually obtained by electrical poling. This technique is based on the application of a strong DC electric field (~10^6^ V/m) at elevated temperatures (~393 K) on the polymer through a top and bottom electrode, in order to induce the alignment of the dipoles along the direction of the electric field [7]. However, electrical poling has some limitations in terms of cost-effectiveness and practical implementation. Recently, several alternative techniques, aimed at improving the nucleation of the electroactive state in PVDF, such as mechanical stretching, heat controlled spin-coating, and phase inversion, have been investigated [8,9,10]. In particular the phase inversion technique was investigated in Reference [10], and the effect of temperature on the piezoelectric coefficient was examined in the 253 K–373 K range. It was demonstrated that at the temperature of 253 K, the β-phase crystallites are self-aligned, and the PDVF film is characterized by a high piezoelectric coefficient. It resulted that self-aligned β-phase PVDF films with high piezoelectric coefficient were produced via phase inversion, through spin-coating and quenching. However, the mechanism for the self-alignment of the β-crystals was not fully understood, due to the competitive role of three different factors: The mechanical stretching of the polymer chains during spinning, the thermal gradient during quenching, the electrostatic interaction between the polymer film and the polar molecules of water.

In References [2,11], the authors investigated the influence on the β-phase formation and the piezoelectric response enhancement of several process parameters. These included the polymer concentration (from 10 to 24 wt.%), the rotational speed ranging from 1000 rpm to 8000 rpm, and the crystallization temperature, in the range from 293 K to 363 K. It was shown that the β-phase increases in general with the rotation speed of the spin-coater, up to a limit value, which increases with the polymer concentration in the solvent. Moreover, the electroactive phase reduces dramatically as the baking temperature increases over 323 K.

The scope of this work is to assess to what extent the mechanical stretching of the polymeric chains, produced by the spin-coater rotation during film deposition, is responsible for the formation of the β-phase and the enhancement of the piezoelectric response.

For this purpose, piezoelectric PVDF thin films were produced through spin-coating and phase inversion by quenching, avoiding the use of electrical poling. For the first time, we investigated the effect of quenching on spin-coated PVDF films at very low temperature (~77 K) in a non-polar medium (i.e., liquid nitrogen) in order to avoid polarization, due to electrostatic interaction or temperature gradient. In particular, we studied how the piezoelectric performance of PVDF films is affected by: (i) The polymer/solvent mass ratio; (ii) the rotational spin speed; and (iii) the quenching temperature. The relative volume fraction of the β-phase (*F*(β)) of the produced films was evaluated through Fourier Transform Infrared (FT-IR) spectroscopy, and the piezoelectric response was assessed through measurements of the coefficient *d*_33_ using Piezoresponse Force Microscopy (PFM). We quantitatively evaluated the *d*_33_ avoiding the use of a top electrode [1] and using the characterization protocol based on PFM described in Reference [12]. The morphology of the sample was evaluated through Field Emission Scanning Electron Microscopy (FE-SEM). The viscosity of the prepared PVDF solutions was measured, before film deposition, through rheology. The obtained results demonstrated that, based on the viscosity of the PVDF solution and on the quenching temperature, the best piezoelectric response is originated either by the electrostatic interaction of the polymer chains with polar molecules of water or by the mechanical stretching induced by the spin-coater’s rotation.

## 2. Materials and Methods

The PVDF samples were obtained by the combination of spin-coating and quenching treatment in order to increase the piezoelectric coefficient *d*_33_ and the electroactive phase *F*(β) content.

The samples were produced as follows: firstly, PVDF in powder form (Solvay 6010, Solvay Specialty Polymers S.P.A., Bollate, Italy) was dissolved in N,N dimethylformamide (DMF ≥99%, Sigma–Aldrich, Saint Louis, MO, USA) by magnetic stirring (Heidolph Instruments, Schwabach, Germany) for 3 h at 343 K. Then, 1 mL of the produced polymer solution was cast over a PET/ITO substrate (R_s_ = 60 Ω/sq., Sigma–Aldrich, Saint Louis, MO, USA) [~(3 × 3) cm^2^] and spin-coated (Semiconductor Production Systems Europe, Putten, Netherlands) with an acceleration of 7500 rpm/s^2^ for 10 s. After the deposition the sample was immersed immediately in an opportune bath (H_2_O; H_2_O+glycerol or liquid N_2_) held at various quenching temperatures (303 K, 253 K or 77 K) for 30 min. 

In this work, we considered the two polymer concentration of 20 wt.% and 30 wt.% Moreover, the films were produced at two different rotation speeds of the spin-coater (i.e 2500 rpm and 7500 rpm) and considering the following three quenching conditions—(i) 303 K in a water bath; (ii) 253 K in an ice/glycerol bath; and (iii) 77 K in a liquid nitrogen bath.

The produced samples are schematically summarized in Table 1. The films produced with 20 wt.% of PVDF at 2500 rpm or 7500 rpm have average thicknesses of approximately (17 ± 3) μm or (7.7 ± 1.5) μm, respectively. For the sample containing 30 wt.% of PDVF, the average thickness is (46 ± 2) μm or (20 ± 2) μm, corresponding to a spinning speed of 2500 rpm or 7500 rpm, respectively.

### Characterization

A Field Emission Scanning Electron Microscope (FE-SEM, Auriga, Carl Zeiss, Oberkochen, Germany) operating with an accelerating voltage of 3 kV was used to assess the morphology of the PVDF films. A Quorum Technologies Q150T ES sputter coater (Laughton, East Sussex, UK) was used to metallize the PVDF films prior to SEM imaging with 20 nm of Cr, in order to prevent charging.

The rheological behaviour of the material was assessed using a Modular Compact Rheometer (MCR302, Anton Paar, Rivoli, Torino). The measurement setup was composed of a 25 mm parallel plate system with 1 mm gap between the plates. Rheological tests were carried out at room temperature, in the steady shear regime. The rheological behaviour of the two different PVDF-DMF solutions was assessed as a function of the shear rate from 0.01 to 100 s^−1^.

FT-IR characterizations were performed as described in References [12,13,14]. In particular, FT-IR measurements were carried out in the 4000–600 cm^−1^ range with a resolution of 1 cm^−1^ (Bruker Billerica, MA, USA).

The piezoelectric coefficient $d_{33}$ was measured through the PFM technique using a commercial Dimension Icon AFM (Bruker-Veeco, Billerica, MA, USA) [13,15]. Following the procedure described in Reference [12], we scanned three different areas (1 × 1) μm^2^ in size on each sample, with 256 × 256 acquisition points per scanning area, and with a scan rate of 0.5 Hz, in order to obtain an estimate of the average value of $d_{33}$, which is representative of the piezoresponse of the sample at macroscale.

The procedure applied for PFM characterization can be summarized as follows:

(i) At first, a calibration measurement of a reference periodically poled lithium niobate (PPLN, Bruker Billerica, MA, USA) sample, with a known value of the piezoresponse coefficient (*d*_33__PPLN_), is conducted over the amplitude voltage range from 2 V to 10 V;
(ii) Then, the sample under investigation is tested, and the amplitude of the PFM signal $V_{\mathrm{PVDF}}$ is measured as a function of the applied voltage $V_{ac}$ in the selected range;
(iii) The PPLN is measured again and the new vertical piezoresponse force microscopy signals (VPFM), for each value of the applied voltage $V_{ac}$, are compared with the corresponding values measured during the first calibration cycle;
(iv) If the difference between the two piezoelectric calibration signals is less than 20%, the measurement of the sample under test is considered reliable;
(v) Next, the calibration factor $\xi =m_{\mathrm{PPLN}}/d_{33\mathrm{PPLN}}$ (in which $m_{\mathrm{PPLN}}$ is the slope of the straight line of the amplitude of the VPFM signal vs. $V_{ac}$, and $d_{33\mathrm{PPLN}}$ is the known piezoelectric coefficient of the PPLN) is evaluated, averaging over the two calibration measurements;
(vi) The *d*_33_ value of the sample under test is finally calculated, using the following expression:
  (1) $$d_{33}=m=\frac{V_{\mathrm{PVDF}}}{\xi \;V_{\mathrm{ac}}},$$
  in which $V_{\mathrm{PVDF}}$ is the vertical displacement of the sample under test.

Once the PFM measurements were performed in each selected areas of the sample, we estimated the average PFM response as:

(2) $$\langle d_{33}\rangle =\left(d_{33}^{1}+d_{33}^{2}+d_{33}^{3}\right)/3,$$

and the highest PFM response as:

(3) $$d_{33max}=\max\left\{d_{33}^{1},d_{33}^{2},d_{33}^{3}\right\}.$$

The standard deviation of $\langle d_{33}\rangle$ is representative of the uniformity of the piezoresponse of the sample. 

## 3. Results

### 3.1. Rheological Characterization

**Figure 1:**
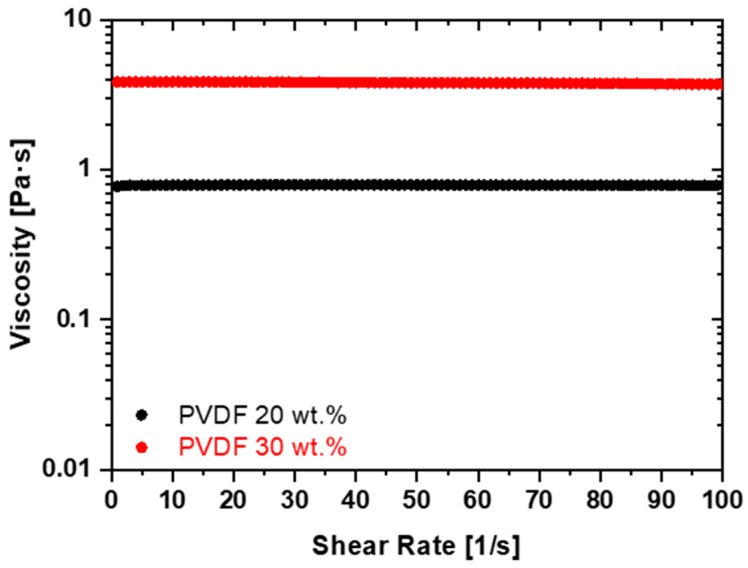
Viscosity curves of PVDF/DMF (N,N dimethylformamide) solutions with different polymer concentrations.

The rheological analysis showed a Newtonian behavior of the produced polymer-DMF solutions for both PVDF concentrations of 20 wt.% and 30 wt.% The increase in the PVDF to solvent ratio produced an increase in viscosity of the solution, regardless of the shear rate, as shown in Figure 1. Moreover, it can be noticed that the material retains the Newtonian behavior even for the highest polymer concentration. The viscosity of the solution with a polymer concentration of 20 wt.% is $\eta_{20\%}=0.79$ Pa·s, while it shows an almost five-fold increase up to the value of $\eta_{30\%}=3.86$ Pa·s for a PVDF content of 30 wt.%. 

On the basis of the rheological characterizations, considering the high values of the spin-coater rotation speed (either 2500 rpm or 7500 rpm), the relatively short deposition time of 10 s and the low volatility of DMF at room temperature (i.e., 0.49 kPa), we can assume that the solvent evaporation is negligible during deposition and that the viscosity of the solution is nearly constant. Therefore, the film thickness h can be estimated using the following expression, according to Reference [16]:

(4) $$h=\frac{h_{0}}{\sqrt{1+\frac{4\rho \omega^{2}h_{0}^{2}t}{3\eta }}},$$

in which ω is the spin-coater angular velocity, t is the deposition time, η and ρ are the dynamic viscosity and density of the polymer solution, respectively, $h_{0}$ is the initial thickness of the film after the starting transient, i.e., when the plate has reached a constant angular speed. The value of $h_{0}$ can be estimated experimentally, and it is mainly affected by the wettability of the substrate, by the PVDF-DMF solution viscosity, by the initial angular acceleration of the plate providing a twisting force to the polymer. On the basis of previous studies [10,11] and using Equation (4), we can estimate that for the 20 wt.% PVDF film, $h_{0}$ is in the range 8–20 μm, whereas for the 30 wt.% PVDF film it is in the range 40–70 μm. The density of the solution, ρ, is calculated using the following expression: $\rho =M_{solution}/V_{solution}$, where $M_{solution}=m_{PVDF}+m_{DMF}$, being $m_{PVDF}$ and $m_{DMF}$ the measured masses of the PVDF and DMF respectively, and $V_{solution}=m_{PVDF}/\rho_{PVDF}+m_{DMF}/\rho_{DMF}$, considering the density of PVDF $\rho_{PVDF}$ = 1.78 g/cm^3^ and the one of DMF $\rho_{DMF}=$ 0.944 g/cm^3^.The calculated densities are: $\rho_{20\%}=$ 1.024 g/cm^3^ and $\rho_{30\%}=$1.060 g/cm^3^. Therefore, from Equation (4) and using the measured values of viscosity, we estimate the thickness of the samples, as reported in Table 2. The estimated thickness values are in good agreement with experimentally-measured ones, obtained with a commercial micrometer (see Table 2). The good agreement guarantees the accuracy of the evaluation of the solution’s density used in Equation (4).

### 3.2. Surface Morphology

**Figure 2:**
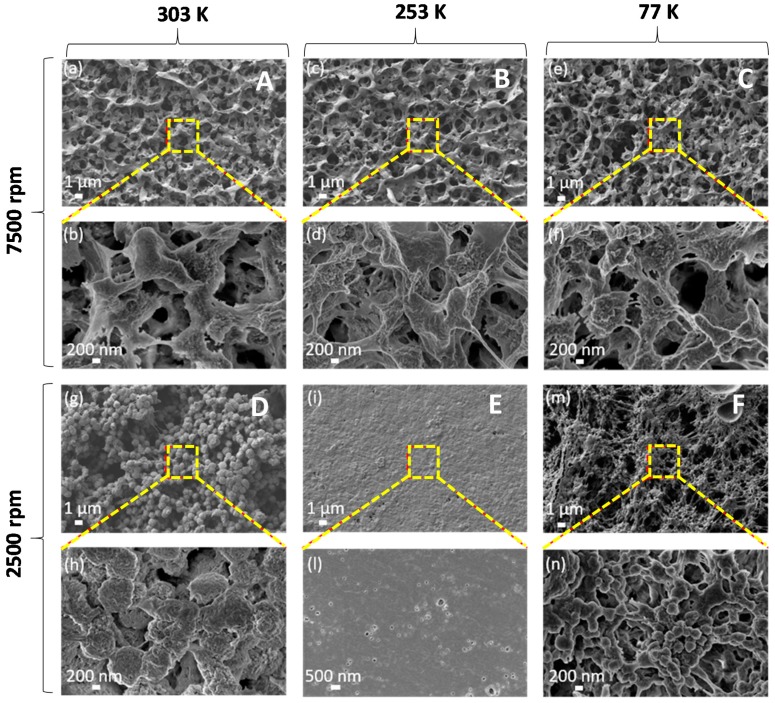
FE-SEM low-magnification (**a**,**c**,**e**,**g**,**i**,**m**) and high-magnification (**b**,**d**,**f**,**h**,**l**,**n**) micrographs of PVDF films with polymer content of 20 wt.%., produced at spin-coater rotation speed of 7500 rpm (samples A, B, C) or 2500 rpm (samples D, E, F), and quenched at 303 K in water (samples A, D) or at 253 K in water and glycerol (samples B, E) or at 77 K in liquid nitrogen (samples C, F).

**Figure 3:**
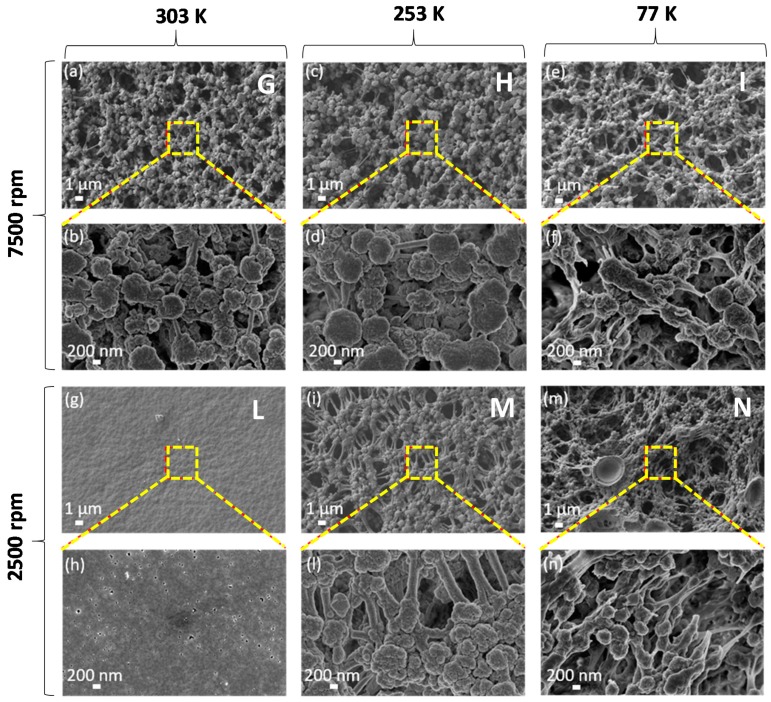
FE-SEM low-magnification (**a**,**c**,**e**,**g**,**i**,**m**) and high-magnification (**b**,**d**,**f**,**h**,**l**,**n**) micrographs of PVDF films with polymer content of 30 wt.%., produced at spin-coater rotation speed of 7500 rpm (samples G, H, I) or 2500 rpm (samples L, M, N), and quenched at 303 K in water (samples G, L) or at 253 K in water and glycerol (samples H, M) or at 77 K in liquid nitrogen (samples I, N).

Figure 2 and Figure 3 show FE-SEM micrographs at low (10 kX) and high (25 kX or 50 kX) magnifications of the samples listed in Table 1 and produced with a concentration of PVDF in the solvent of 20 wt.% and the 30 wt.%, respectively.

The produced samples are generally characterized by a porous spherulitic structure, which is originated by solvent evaporation. Size and morphology of the spherulites observed over the surface of the samples are significantly influenced by both spin-coating rotation speed and quenching process.

In particular, it was observed that among the samples produced with the 20 wt.% of PVDF (Figure 2), the ones deposited with an angular speed of 7500 rpm are characterized by a high porosity; spherulites over the surface of these film are evident only in sample A, which was quenched in water, at room temperature. This suggests that spherulites are mainly produced by the effect of electrostatic interaction with the polar water molecules than by the spinning.

As regards the films produced with a PVDF content of 30 wt.% (Figure 3), we noticed that they are all characterized by a spherulitic structure. From the SEM images, we estimated the mean value of the spherulite diameter using an image processing software (ImageJ ©, National Institute of Health, Bethesda, MD, USA) and averaging the diameters of ten different spherulites. The obtained values, reported in Table 1 and demonstrated in this article, show spherulites have minimum dimensions for the samples I and N, which have been quenched in liquid N_2_ at 77 K. We also observed that samples produced at a lower angular speed (2500 rpm) and quenched at lower temperature (i.e., 253 K and 77 K) are characterized by a more evident stretching and alignment of the polymer chain along the substrate than the ones produced at higher speed (7500 rpm). In particular, the length of the polymeric filaments is limited to ~1 μm in samples H and I, produced at 7500 rpm; whereas, they reach several microns in samples M and N, produced at 2500 rpm. Moreover, the averaged diameter of such polymeric filaments is around 300–400 nm in sample M and it reduces to 100–200 nm in sample N quenched in liquid nitrogen at 77 K.

### 3.3. FT-IR Analysis

**Figure 4:**
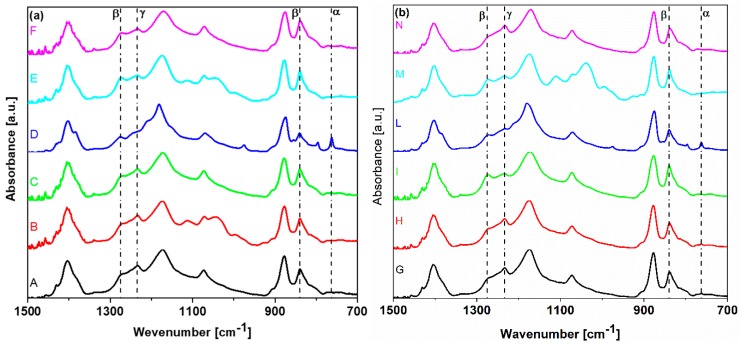
FT-IR spectra of 12 PVDF solutions.

The results of FTIR characterizations are reported in Figure 4a,b. All produced samples exhibit the presence of the β-phase, as highlighted from the characteristic peaks at 840 cm^−1^ and at 1275 cm^−1^. The α-phase is generally present in a low amount, as underlined from the relative amplitude of the characteristic peaks, located in the range between 1423 and 763 cm^−1^ [17,18,19]. The presence of the γ-phase is demonstrated from the presence of the peak at 1234 cm^−1^.

The relative volume fraction of the β-phase F(β) of the produced samples was estimated from the values $A_{\alpha }$ and $A_{\beta }$ of the absorbance at the wavelengths (763 and 840 cm^−1^) associated to the main peaks of the α-and β-phases, respectively, using the following formula [20]:

(5) $$F\left(\beta \right)=\frac{A_{\beta }}{\left(K_{\beta }/K_{\alpha }\right)A_{\alpha }+A_{\beta }},$$

in which it is assumed that the ratio between the absorption coefficients of the α- and β-phases is $K_{\beta }/K_{\alpha }\sim 1.3$.

The obtained values of F(β) are reported in Table 1. The highest value of F(β) (i.e., 97.82%) was observed in sample N, which was produced dissolving 30 wt.% of PVDF, using a quenching temperature of 77 K and a spinning speed of 2500 rpm. It can be noticed that the obtained values are comparable to those reported in the literature for PVDF thin films obtained using a phase inversion technique through spin-coating and quenching [2,10,11].

### 3.4. PFM Characterization

**Figure 5:**
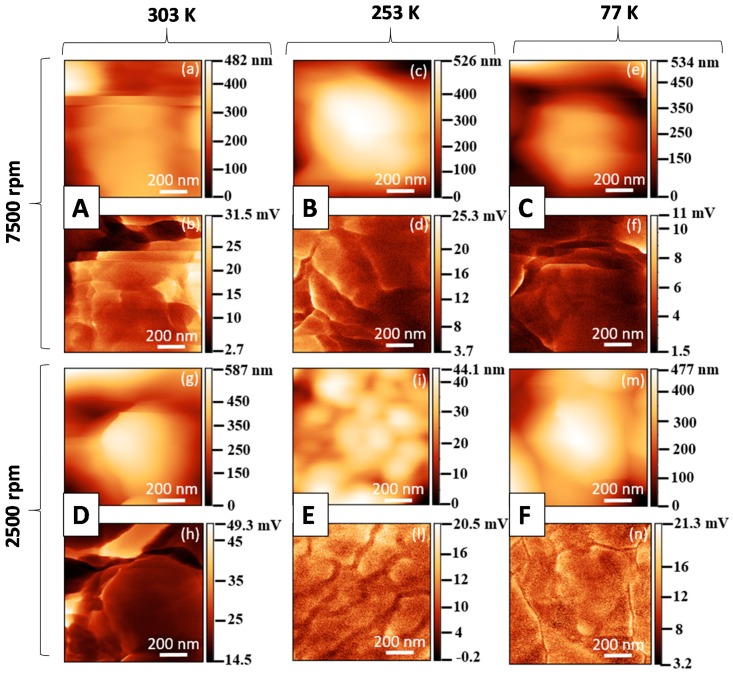
Morphological maps and Piezoresponse Force Microscopy (PFM) signals at V_ac_ = 10 V and at 15 kHz for PVDF at 20 wt.% produced using different temperatures and spinning speeds: (**a**,**b**) Sample A, (**c**,**d**) sample B, (**e**,**f**) sample C, (**g**,**h**) sample D, (**i**,**l**) sample E, (**m**,**n**) sample F.

**Figure 6:**
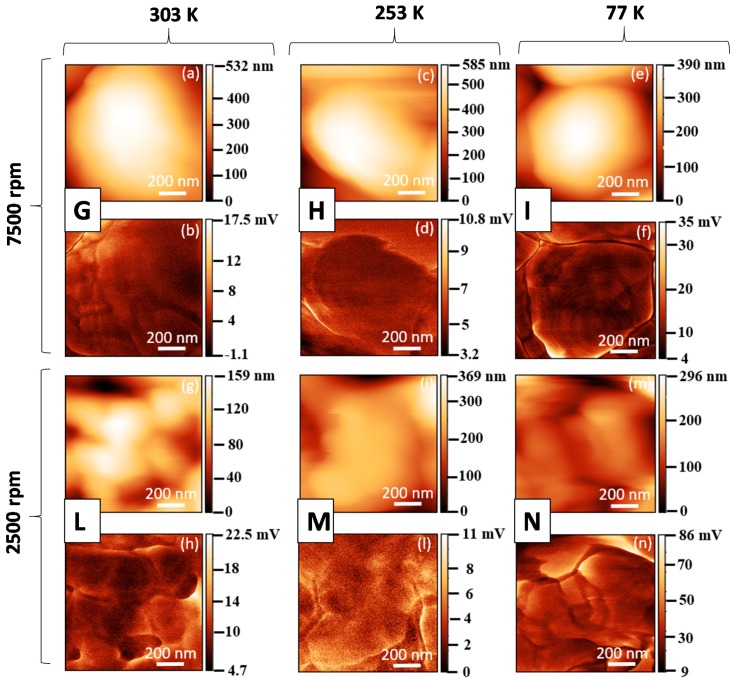
Morphological maps and PFM signals at V_ac_ = 10 V and at 15 kHz for PVDF at 30 wt.% produced at different temperatures and spinning speeds: (**a**,**b**) Sample G, (**c**,**d**) sample H, (**e**,**f**) sample I, (**g**,**h**) sample L, (**i**,**l**) sample M, (**m**,**n**) sample N.

**Figure 7:**
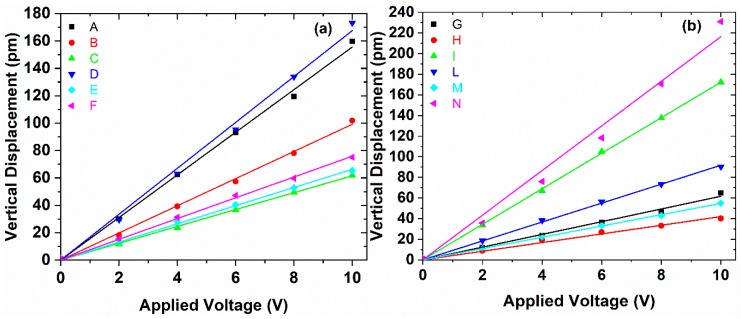
The average amplitude of the vertical displacement, measured through PFM as a function of the applied voltage $V_{ac}$: For the PVDF samples at 20 wt.% at different temperatures and spinning speeds (**a**) and for the PVDF samples at 30 wt.% at different temperature and spinning speed (**b**).

**Figure 8:**
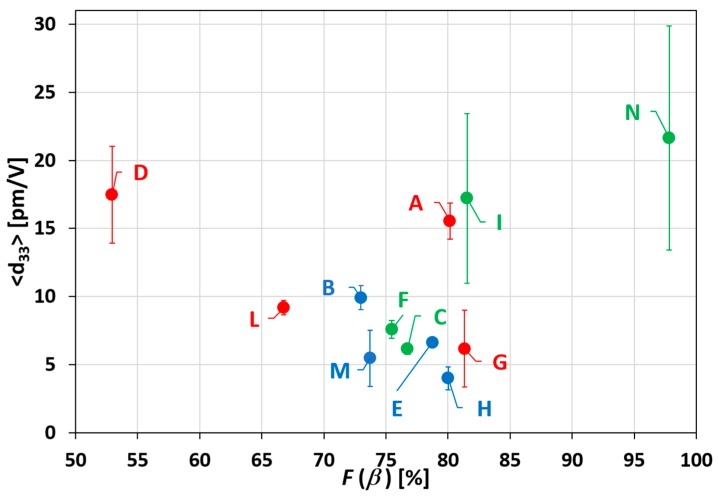
Results of the averaged d_33_ as a function of F(β) of all produced samples, with standard deviation.

The morphological maps and the vertical PFM signals over a scan area (1 × 1) μm^2^ are reported in Figure 5 and Figure 6 for all produced samples of Table 1. The average value $\langle d_{33}\rangle$ was obtained, according to the procedure described in Section 2.1, from the slope of the linear fitting of the VPFM as a function of *V_ac_* (Figure 7). The values of $\langle d_{33}\rangle$, with the corresponding standard deviations and of the maximum value of $d_{33}$, are also included in Table 1 and reported in Figure 8 as a function of F(β) [12,14].

It is interesting to observe that the samples produced at 253 K show lower piezoelectric coefficient, although the *F*(β) is always higher than 70%. Furthermore, among the samples quenched at 303 K (i.e., A, D, G, L), the ones containing the lower polymer concentration (i.e., samples A and D), show a better piezoresponse. On the contrary, if the samples are quenched at 77 K, the higher piezoelectric response is obtained when the highest polymer concentration (30 wt.%) is used samples (L, N). The highest value of $\langle d_{33}\rangle =\left(21.64\pm 8.23\right)\;\mathrm{pm}/V$ and of $d_{33\max}=29.87$ pm/V was measured for the sample N, which is also characterized by the highest value of *F*(β) = 97.82%.

## 4. Discussion

Electroactive phase formation in PVDF films produced through spin-coating and quenching has been investigated in order to assess the main factors that affect phase inversion. In fact, several studies highlighted that β-phase formation in PVDF films is the result of the action of several factors, such as [2,10,11]: (i) The mechanical stretching of the polymeric chains induced during spin-coating; (ii) the thermal gradient acting on the deposited film during quenching in a water bath below 0 °C; (iii) the electrostatic interaction between the CF_2_–CH_2_ groups of PVDF and polar molecules of water.

In this study, we demonstrated that the aforementioned three factors do not always contribute effectively to the enhancement of the piezoelectric properties of the material. Moreover, depending on the selected spin-coating condition, PVDF concentration in the initial DMF solution, and expected thickness of the film, it is possible to choose the proper quenching treatment enabling to achieve the best enhancement of the piezoelectric properties of the material. 

For this purpose, we considered three different quenching conditions:

(1) Quenching at room temperature in water: The electrostatic interaction with the polar molecules of H_2_O is the dominant polarization factor during polymer crystallization;
(2) Quenching at 253 K in a water/glycerol solution: The temperature gradient across the sample combined with the electrostatic interaction with water molecules are the main polarization factors acting during polymer crystallization;
(3) Quenching at 77 K in liquid nitrogen: Neither the temperature gradient, nor the electrostatic interaction, are present. In this case, quenching has only the function of producing an instantaneous crystallization of the polymer, so that mechanical relaxation after spin-coating deposition is inhibited.

Therefore, from the combined analysis of the morphological, FTIR and PFM investigations, we can draw the following conclusions.

The best stretching and alignment of the polymeric chains along the substrate is obtained producing the film by spin-coating at a lower angular speed (i.e., 2500 rpm). During spin-coating deposition, due to the very different molecular mass of fluorine and hydrogen, the centrifugal force induces orientation of the polar groups of CF_2_ and CH_2_ in PVDF. Therefore, the rapid freezing of the PVDF film at very low temperature in liquid nitrogen, which is an apolar molecule, produces an instantaneous crystallization of the material, thus preventing elastic relaxation of the polymeric chains. This phenomenon is the dominant one, especially for the case of solutions with a higher viscosity, containing 30 wt.% of PVDF. This is confirmed by the high value of β-phase content, by the high value of measured piezoresponse coefficient, and by the formation of spherulites over the sample surface, interconnected by polymeric filaments that have a diameter below 200 nm, and lengths exceeding a few microns.

In the case of a more dilute solution, made with PDVF solution of 20 wt.%, the effect of mechanical stretching during spin-coating is limited, due to the low viscosity of the polymeric solution. Actually, samples with 20 wt.% of PVDF quenched at 77 K in liquid nitrogen, were characterized by a low piezoresponse coefficient. This confirms that β-phase crystallization is produced essentially through the electrostatic interaction of PVDF polar groups with polar molecules of water, occurring during quenching in a water bath at room temperature (303 K). In fact, the mechanism that is believed to be responsible for the increase of the piezoelectric coefficient in the sample at 20 wt.% of PVDF quenched at 303 K, is the formation of hydrogen bonds between the O–H groups of water with C–H groups of PVDF. The formation of these hydrogen bonds induce the PVDF molecules on the water to be oriented with C–F groups downwards to water, as described in References [10,21]. The contextual action of a thermal gradient originated by cooling in a bath of water and glycerol (i.e., quenching at 253 K) is not constructive with respect to the effect of electrostatic interaction. In fact, we observed that the highest value of piezoresponse was measured in samples at 20 wt.% of PVDF, quenched in water at room temperature. These samples are also characterized by spherulitic structures, due to the solid–liquid phase separation that occurs during the crystallization phase [12,13,14,22], with an average spherulite diameter in the range 700–800 nm. Differently, from the samples produced with PVDF at 30 wt.%, the formation of polymer filaments is not observed over the film surface: This confirms that the effect of mechanical stretching produced by spin-coating deposition is not the dominant factor in the enhancement of the piezoresponse.

**Figure 9:**
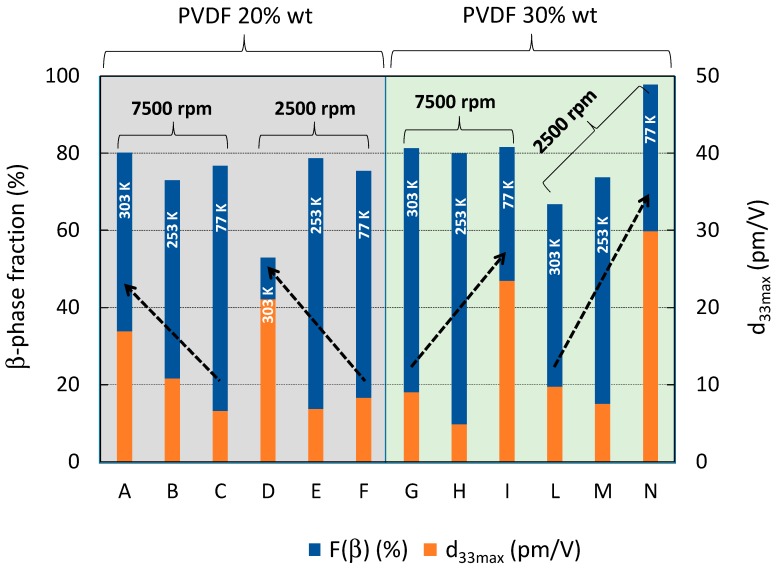
β-phase relative volume fraction and maximum measured piezoresponse coefficient of all produced samples.

From the histogram of Figure 9, it is evident that the increase of the piezoresponse of the material is obtained through quenching at the highest temperature of 303 K of the film produced with the less concentrated PVDF solution. On the contrary, it is achieved through quenching at the lowest temperature of 77 K in the case of films produced by solution with the highest concentration of PVDF.

## 5. Conclusions

In this paper, we demonstrated that the process that enables to obtain films with the highest β-phase and piezoresponse coefficient depends on the initial concentration of PVDF over DMF. In fact, the viscosity of the polymer solution plays a crucial role in the orientation of the polymer chains, due to mechanical stretching induced by spinning.

FT-IR and PFM data, in some cases, do not have the same trend as a function of the quenching temperature. This is due to the fact that although the FT-IR measurements give us information about the amount of β-phase in the sample, nevertheless they do not give us any information about the orientation of the polymeric chains. In any case, it is important to underline that the highest *d*_33_ is associated with the highest amount of F(β). 

The samples quenched at 303 K showed the best *d*_33_ among the samples produced with 20 wt.% of PVDF, indicating that the dominant factor in phase inversion is the electrostatic interaction between the polar groups of PVDF and water molecules. For the samples with a polymer/solvent mass ratio of 30 wt.%, the best *d*_33_ is obtained using a quenching temperature of 77 K, indicating a better orientation and alignment of the polymer chains, which are frozen using liquid N_2_. 

In this work, the combination of the deposition of thin films through the spin process, and the phase inversion through a quenching process, gave us a considerable enhancement of the *d*_33_ if compared with our previous studies [12,13,14,23], as well as with works reported in the literature for PVDF composites and other piezoelectric materials [7,8,9,10,24,25,26,27,28,29]. 

The combination of spin-coating and phase inversion opensspi a new way to realize highly efficient piezoelectric PVDF thin films with an enhanced *d*_33_, without the need of electrical poling or mechanical stretching, suitable to realize devices for energy harvesting and wearable sensors.

## Figures and Tables

**Table 1:** List of the produced specimens of Poly(vinylidene fluoride) (PVDF) thin films, production conditions and piezoelectric properties.

Sample	PVDF Concentr. (wt.%)	Spinning Speed (rpm)	Quenching Temperature (K)	Quenching Medium	Spherulite Diameter (nm)	F(β) (%)	$\langle d_{33}\rangle$ (pm/V)	*d*_33max_ (pm/V)
A	20	7500	303	H_2_O	754 ± 110	80	15.55± 1.32	16.87
B	20	7500	253	H_2_O+glycerol	-	73	9.92 ± 0.87	10.79
C	20	7500	77	N_2_	-	77	6.16 ± 0.41	6.57
D	20	2500	303	H_2_O	761 ± 130	53	17.47 ± 3.56	21.03
E	20	2500	253	H_2_O+glycerol	-	79	6.63 ± 0.22	6.85
F	20	2500	77	N_2_	268 ± 44	75	7.58 ± 0.67	8.25
G	30	7500	303	H_2_O	609 ± 89	81	6.17 ± 2.83	9.00
H	30	7500	253	H_2_O+glycerol	804 ± 137	80	3.99 ± 0.85	4.84
I	30	7500	77	N_2_	575 ± 90	81	17.22 ± 6.24	23.46
L	30	2500	303	H_2_O	-	67	9.17 ± 0.52	9.69
M	30	2500	253	H_2_O+glycerol	609 ± 116	74	5.47 ± 2.06	7.53
N	30	2500	77	N_2_	386 ± 61	98	21.64 ± 8.23	29.87

**Table 2:** Measured and estimated thickness for the produced samples.

Sample	PVDF Concentr. (wt.%)	Spinning Speed (rpm)	Measured Thickness (μm)	Estimated Thickness from Equation (4) (μm)
A/B/C	20	7500	7.7 ± 1.5	7
D/E/F	20	2500	17 ± 3.0	16
G/H/I	30	7500	20 ± 2.0	18
L/M/N	30	2500	46.0 ± 2.0	45
